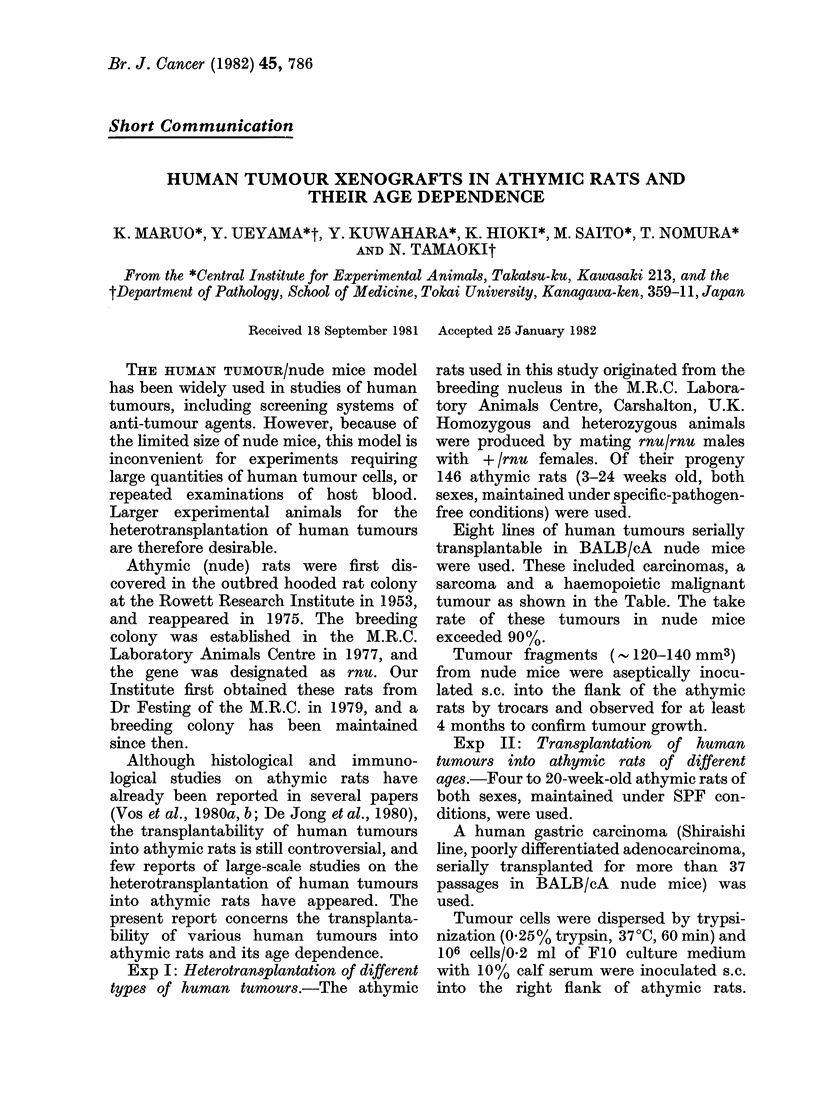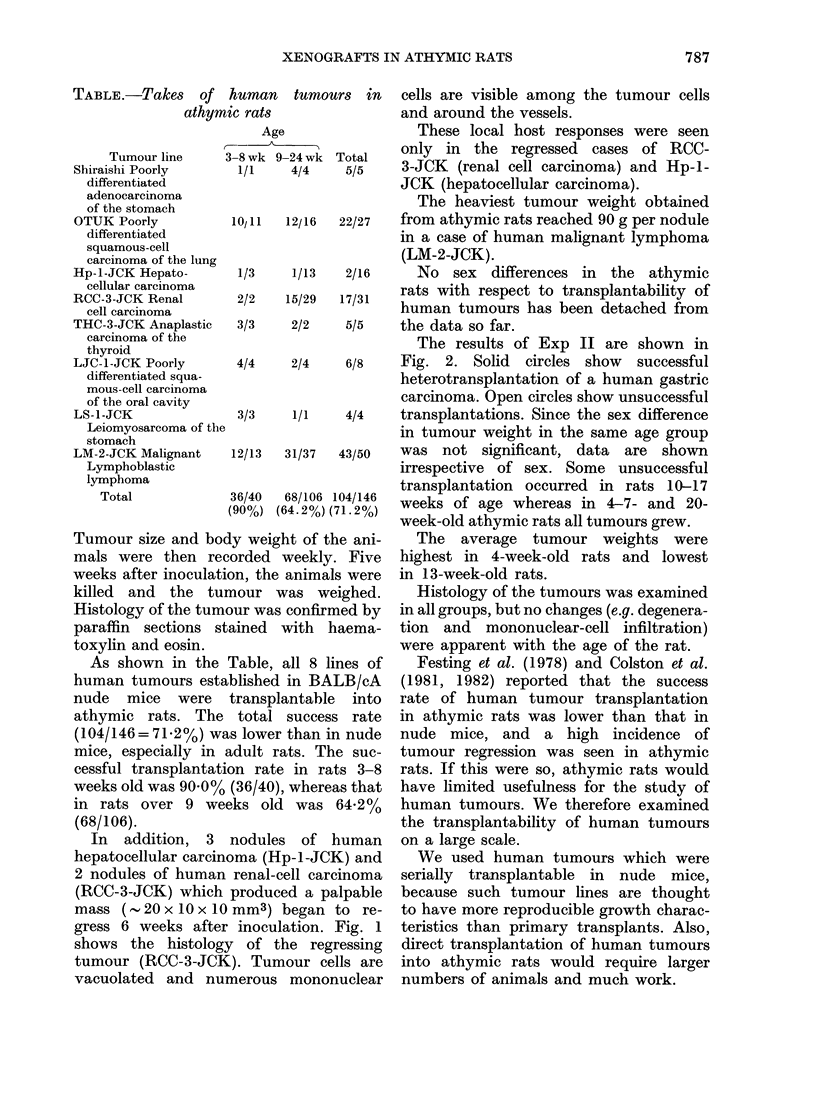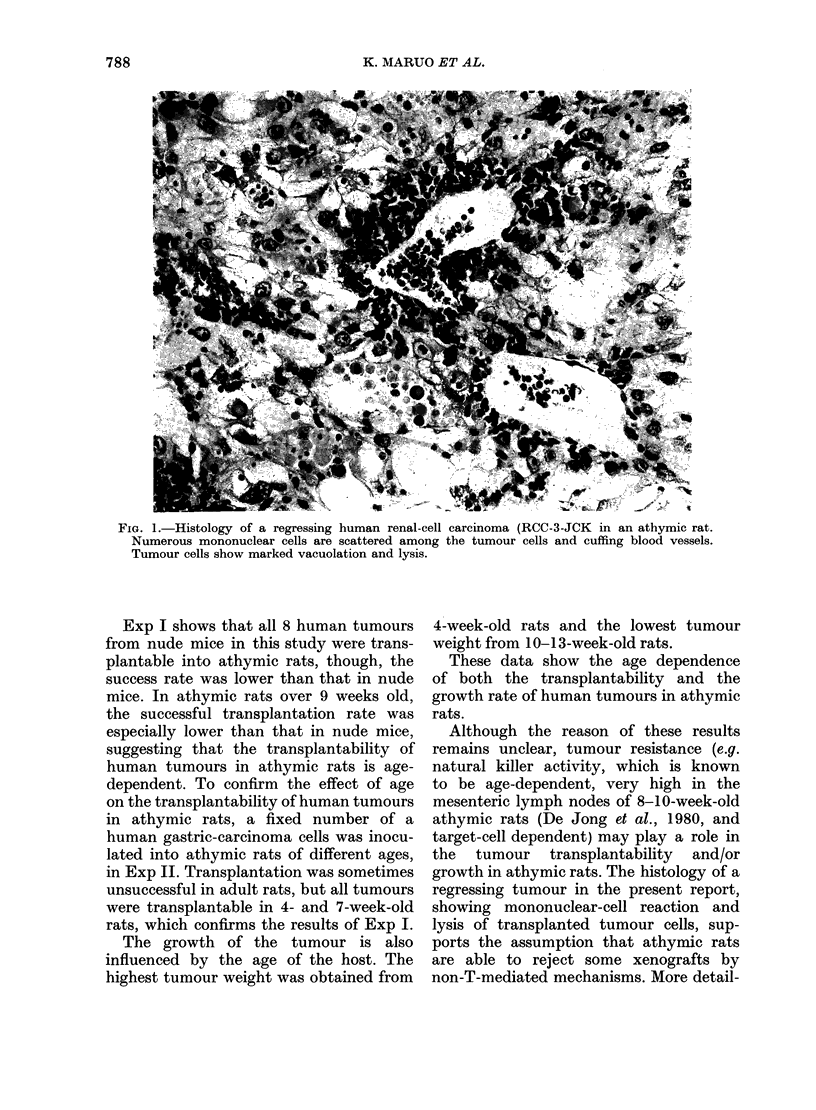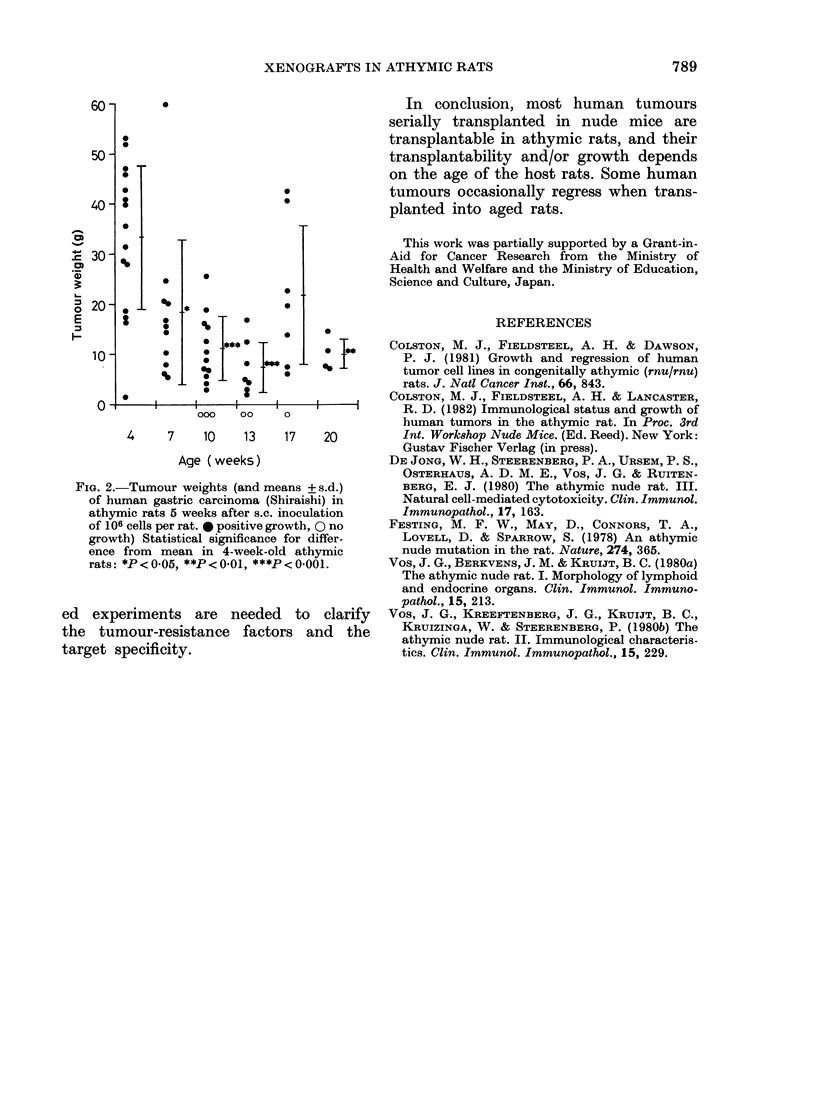# Human tumour xenografts in athymic rats and their age dependence.

**DOI:** 10.1038/bjc.1982.122

**Published:** 1982-05

**Authors:** K. Maruo, Y. Ueyama, Y. Kuwahara, K. Hioki, M. Saito, T. Nomura, N. Tamaoki

## Abstract

**Images:**


*Br. J. Cancer* (1982) **45**, 786

***Short Communication***

### HUMAN TUMOUR XENOGRAFTS IN ATHYMIC RATS AND THEIR AGE DEPENDENCE

K. MARUO*, Y. UEYAMA*†, Y. KUWAHARA*, K. HIOKI*, M. SAITO*, T. NOMURA* AND N. TAMAOKI†

*From the *Central Institute for Experimental Animals, Takatsu-ku, Kawasaki* 213, *and the †Department of Pathology, School of Medicine, Tokai University, Kanagawa-ken*, 359–11, *Japan*

Received 18 September 1981 Accepted 25 January 1982

THE HUMAN TUMOUR/nude mice model has been widely used in studies of human tumours, including screening systems of anti-tumour agents. However, because of the limited size of nude mice, this model is inconvenient for experiments requiring large quantities of human tumour cells, or repeated examinations of host blood. Larger experimental animals for the heterotransplantation of human tumours are therefore desirable.

Athymic (nude) rats were first discovered in the outbred hooded rat colony at the Rowett Research Institute in 1953, and reappeared in 1975. The breeding colony was established in the M.R.C. Laboratory Animals Centre in 1977, and the gene was designated as *rnu*. Our Institute first obtained these rats from Dr Festing of the M.R.C. in 1979, and a breeding colony has been maintained since then.

Although histological and immunological studies on athymic rats have already been reported in several papers (Vos *et al.*, 1980*a*, *b*; De Jong *et al.*, 1980), the transplantability of human tumours into athymic rats is still controversial, and few reports of large-scale studies on the heterotransplantation of human tumours into athymic rats have appeared. The present report concerns the transplantability of various human tumours into athymic rats and its age dependence.

Exp I: *Heterotransplantation of different types of human tumours.*—The athymic rats used in this study originated from the breeding nucleus in the M.R.C. Laboratory Animals Centre, Carshalton, U.K. Homozygous and heterozygous animals were produced by mating *rnu/rnu* males with +/*rnu* females. Of their progeny 146 athymic rats (3–24 weeks old, both sexes, maintained under specific-pathogen-free conditions) were used.

Eight lines of human tumours serially transplantable in BALB/cA nude mice were used. These included carcinomas, a sarcoma and a haemopoietic malignant tumour as shown in the Table. The take rate of these tumours in nude mice exceeded 90%.

Tumour fragments (~120–140 $mm^3$) from nude mice were aseptically inoculated s.c. into the flank of the athymic rats by trocars and observed for at least 4 months to confirm tumour growth.

Exp II: *Transplantation of human tumours into athymic rats of different ages.*—Four to 20-week-old athymic rats of both sexes, maintained under SPF conditions, were used.

A human gastric carcinoma (Shiraishi line, poorly differentiated adenocarcinoma, serially transplanted for more than 37 passages in BALB/cA nude mice) was used.

Tumour cells were dispersed by trypsinization (0·25% trypsin, 37°C, 60 min) and $10^6$ cells/0·2 ml of F10 culture medium with 10% calf serum were inoculated s.c. into the right flank of athymic rats.

TABLE.—*Takes of human tumours in athymic rats*

| Tumour line | Age 3–8 wk | Age 9–24 wk | Total |
|---|---|---|---|
| Shiraishi Poorly differentiated adenocarcinoma of the stomach | 1/1 | 4/4 | 5/5 |
| OTUK Poorly differentiated squamous-cell carcinoma of the lung | 10/11 | 12/16 | 22/27 |
| Hp-1-JCK Hepato-cellular carcinoma | 1/3 | 1/13 | 2/16 |
| RCC-3-JCK Renal cell carcinoma | 2/2 | 15/29 | 17/31 |
| THC-3-JCK Anaplastic carcinoma of the thyroid | 3/3 | 2/2 | 5/5 |
| LJC-1-JCK Poorly differentiated squamous-cell carcinoma of the oral cavity | 4/4 | 2/4 | 6/8 |
| LS-1-JCK Leiomyosarcoma of the stomach | 3/3 | 1/1 | 4/4 |
| LM-2-JCK Malignant Lymphoblastic lymphoma | 12/13 | 31/37 | 43/50 |
| Total | 36/40 (90%) | 68/106 (64.2%) | 104/146 (71.2%) |

Tumour size and body weight of the animals were then recorded weekly. Five weeks after inoculation, the animals were killed and the tumour was weighed. Histology of the tumour was confirmed by paraffin sections stained with haematoxylin and eosin.

As shown in the Table, all 8 lines of human tumours established in BALB/cA nude mice were transplantable into athymic rats. The total success rate (104/146 = 71·2%) was lower than in nude mice, especially in adult rats. The successful transplantation rate in rats 3–8 weeks old was 90·0% (36/40), whereas that in rats over 9 weeks old was 64·2% (68/106).

In addition, 3 nodules of human hepatocellular carcinoma (Hp-1-JCK) and 2 nodules of human renal-cell carcinoma (RCC-3-JCK) which produced a palpable mass ($\sim 20 \times 10 \times 10$ mm$^3$) began to regress 6 weeks after inoculation. Fig. 1 shows the histology of the regressing tumour (RCC-3-JCK). Tumour cells are vacuolated and numerous mononuclear cells are visible among the tumour cells and around the vessels.

These local host responses were seen only in the regressed cases of RCC-3-JCK (renal cell carcinoma) and Hp-1-JCK (hepatocellular carcinoma).

The heaviest tumour weight obtained from athymic rats reached 90 g per nodule in a case of human malignant lymphoma (LM-2-JCK).

No sex differences in the athymic rats with respect to transplantability of human tumours has been detached from the data so far.

The results of Exp II are shown in Fig. 2. Solid circles show successful heterotransplantation of a human gastric carcinoma. Open circles show unsuccessful transplantations. Since the sex difference in tumour weight in the same age group was not significant, data are shown irrespective of sex. Some unsuccessful transplantation occurred in rats 10–17 weeks of age whereas in 4–7- and 20-week-old athymic rats all tumours grew.

The average tumour weights were highest in 4-week-old rats and lowest in 13-week-old rats.

Histology of the tumours was examined in all groups, but no changes (*e.g.* degeneration and mononuclear-cell infiltration) were apparent with the age of the rat.

Festing *et al.* (1978) and Colston *et al.* (1981, 1982) reported that the success rate of human tumour transplantation in athymic rats was lower than that in nude mice, and a high incidence of tumour regression was seen in athymic rats. If this were so, athymic rats would have limited usefulness for the study of human tumours. We therefore examined the transplantability of human tumours on a large scale.

We used human tumours which were serially transplantable in nude mice, because such tumour lines are thought to have more reproducible growth characteristics than primary transplants. Also, direct transplantation of human tumours into athymic rats would require larger numbers of animals and much work.

Fig. 1.—Histology of a regressing human renal-cell carcinoma (RCC-3-JCK in an athymic rat. Numerous mononuclear cells are scattered among the tumour cells and cuffing blood vessels. Tumour cells show marked vacuolation and lysis.

Exp I shows that all 8 human tumours from nude mice in this study were transplantable into athymic rats, though, the success rate was lower than that in nude mice. In athymic rats over 9 weeks old, the successful transplantation rate was especially lower than that in nude mice, suggesting that the transplantability of human tumours in athymic rats is age-dependent. To confirm the effect of age on the transplantability of human tumours in athymic rats, a fixed number of a human gastric-carcinoma cells was inoculated into athymic rats of different ages, in Exp II. Transplantation was sometimes unsuccessful in adult rats, but all tumours were transplantable in 4- and 7-week-old rats, which confirms the results of Exp I.

The growth of the tumour is also influenced by the age of the host. The highest tumour weight was obtained from 4-week-old rats and the lowest tumour weight from 10–13-week-old rats.

These data show the age dependence of both the transplantability and the growth rate of human tumours in athymic rats.

Although the reason of these results remains unclear, tumour resistance (*e.g.* natural killer activity, which is known to be age-dependent, very high in the mesenteric lymph nodes of 8–10-week-old athymic rats (De Jong *et al.*, 1980, and target-cell dependent) may play a role in the tumour transplantability and/or growth in athymic rats. The histology of a regressing tumour in the present report, showing mononuclear-cell reaction and lysis of transplanted tumour cells, supports the assumption that athymic rats are able to reject some xenografts by non-T-mediated mechanisms. More detail-

FIG. 2.—Tumour weights (and means ± s.d.) of human gastric carcinoma (Shiraishi) in athymic rats 5 weeks after s.c. inoculation of $10^6$ cells per rat. ● positive growth, ○ no growth) Statistical significance for difference from mean in 4-week-old athymic rats: $^*P < 0.05$, $^{**}P < 0.01$, $^{***}P < 0.001$.

ed experiments are needed to clarify the tumour-resistance factors and the target specificity.

In conclusion, most human tumours serially transplanted in nude mice are transplantable in athymic rats, and their transplantability and/or growth depends on the age of the host rats. Some human tumours occasionally regress when transplanted into aged rats.

This work was partially supported by a Grant-in-Aid for Cancer Research from the Ministry of Health and Welfare and the Ministry of Education, Science and Culture, Japan.

### REFERENCES

COLSTON, M. J., FIELDSTEEL, A. H. & DAWSON, P. J. (1981) Growth and regression of human tumor cell lines in congenitally athymic (*rnu/rnu*) rats. *J. Natl Cancer Inst.*, **66**, 843.

COLSTON, M. J., FIELDSTEEL, A. H. & LANCASTER, R. D. (1982) Immunological status and growth of human tumors in the athymic rat. In *Proc. 3rd Int. Workshop Nude Mice.* (Ed. Reed). New York: Gustav Fischer Verlag (in press).

DE JONG, W. H., STEERENBERG, P. A., URSEM, P. S., OSTERHAUS, A. D. M. E., VOS, J. G. & RUITENBERG, E. J. (1980) The athymic nude rat. III. Natural cell-mediated cytotoxicity. *Clin. Immunol. Immunopathol.*, **17**, 163.

FESTING, M. F. W., MAY, D., CONNORS, T. A., LOVELL, D. & SPARROW, S. (1978) An athymic nude mutation in the rat. *Nature*, **274**, 365.

VOS, J. G., BERKVENS, J. M. & KRUIJT, B. C. (1980*a*) The athymic nude rat. I. Morphology of lymphoid and endocrine organs. *Clin. Immunol. Immunopathol.*, **15**, 213.

VOS, J. G., KREEFTENBERG, J. G., KRUIJT, B. C., KRUIZINGA, W. & STEERENBERG, P. (1980*b*) The athymic nude rat. II. Immunological characteristics. *Clin. Immunol. Immunopathol.*, **15**, 229.